# Tim-4 Inhibits NO Generation by Murine Macrophages

**DOI:** 10.1371/journal.pone.0124771

**Published:** 2015-04-23

**Authors:** Li-yun Xu, Jian-ni Qi, Xiao Liu, Hong-xin Ma, Wei Yuan, Pei-qing Zhao, Xiao-hong Liang, Yong Xu, Hong-xing Wang, Xiao-yan Xu, Wei Wang, Chun-hong Ma, Li-fen Gao

**Affiliations:** 1 Department of Immunology, Key Laboratory for Experimental Teratology of Ministry of Education, Shandong Provincial Key Laboratory of Infection & Immunology, Shandong University School of Medicine, 44 Wenhua Xi Road, Jinan, Shandong, 250012, P.R. China; 2 Cell and Molecular Biology Laboratory, Zhoushan Hospital, 739 Dingshen Road, Zhoushan, Zhejiang, 316000, P.R. China; 3 Department of bone surgery, Zhangqiu Hospital, 1920 Huiquan Road, Jinan, Shandong, 250200, P.R. China; Karolinska Institutet, SWEDEN

## Abstract

**Objective:**

T cell immunoglobulin- and mucin-domain-containing molecule-4 (Tim-4) receives much attention as a potentially negative regulator of immune responses. However, its modulation on macrophages has not been fully elucidated so far. This study aimed to identify the role of Tim-4 in nitric oxide (NO) modulation.

**Methods:**

Macrophages were stimulated with 100 ng/ml LPS or 100 U/ml IFN-γ. RT-PCR was performed to detect *TIM-4* mRNA expression. Tim-4 blocking antibody and NF-κB inhibitory ligand were involved in the study. NO levels were assayed by Griess reaction. Phosphorylation of NF-κB, Jak2 or Stat1 was verified by western blot.

**Results:**

Tim-4 was up-regulated in murine macrophages after interferon-gamma (IFN-γ) stimulation. Tim-4 over-expression decreased NO production and inducible nitric oxide synthase (iNOS) expression in lipopolysaccharide (LPS) or IFN-γ-stimulated macrophages. Consistently, Tim-4 blockade promoted LPS or IFN-γ-induced NO secretion and iNOS expression. Tim-4 over-expression decreased LPS-induced nuclear factor kappa B (NF-κB) p65 phosphorylation in macrophages, which was abrogated by NF-κB inhibitory ligand. On the contrary, Tim-4 blocking increased LPS-induced NF-κB signaling, which was also abrogated by NF-κB inhibition. In addition, Tim-4 blockade promoted Jak2 and Stat1 phosphorylation in IFN-γ stimulated macrophages.

**Conclusion:**

These results indicate that Tim-4 is involved in negative regulation of NO production in macrophages, suggesting the critical role of Tim-4 in immune related diseases.

## Introduction

In mammals, nitric oxide (NO) is an important cellular messenger molecule involved in many physiological and pathological processes [1, 2]. Low levels of NO are very important in protecting the organs such as liver from ischemic damage. However, chronic expression of NO is associated with various carcinomas and inflammatory conditions including juvenile diabetes, multiple sclerosis, arthritis, and ulcerative colitis [3–5]. NO, as free radicals, is secreted by phagocytes (monocytes, macrophages, and neutrophils) in the process of immune response and is toxic to bacteria. Phagocytes are armed with inducible nitric oxide synthase (iNOS) [6–8], which is activated by lipopolysaccharide (LPS), interferon-gamma (IFN-γ) or other cytokines. NO is also involved in the human immune responses. Therefore, it is necessary to explore the mechanism of NO modulation.

T cell immunoglobulin- and mucin-domain-containing molecule-4 (Tim-4, also known as Timd4) is a member of TIM family which is a relatively newly described group of molecules and receives much attention as potential regulators of the immune responses. Tim-4 is specifically expressed on macrophages and dendritic cells, but not on T cells [9, 10]. Recently, it is identified to be expressed on NKT cells, mast cells and B1 cells. Tim-4, as one of natural ligands of Tim-1, shows bimodal regulation on T cell-mediated immune responses by interaction with Tim-1, which is selectively expressed on Th2 cells [10]. It has been demonstrated that NO production is increased in the macrophage cell line RAW264.7 with Tim-1 protein stimulation [11]. Nevertheless, it is unclear whether the NO production regulated by Tim-1 is mediated by its interaction with Tim-4. In addition, increasing evidences show that Tim-4 could recognize and combine with phosphatidylserine (PS) on the surface of apoptotic cells, and promote the engulfment of apoptotic cells by macrophages [12, 13], suggesting that Tim-4 might regulate the function of macrophages by unknown signaling pathways. Our published data showed that Tim-4 over-expression could inhibit the expression of MHC-II, CD80, CD86 and the production of TNF-α in LPS-activated murine macrophages [14]. However, the effect of Tim-4 on NO production in activated macrophages remains unknown so far. In this study, we evaluated the effect of Tim-4 on NO production in LPS- and IFN-γ- activated murine macrophages and further explored the signaling pathways.

## Materials and Methods

### Mice

Male BALB/c mice, 6–8 week-old, were purchased from the Laboratory Animal Center of Shandong University. All Mice were housed in the Animal Facilities under specific pathogen-free conditions. This study has been approved by the Animal Care and Use Committee of Shandong University.

### Cell culture

For Tim-4 blockage, peritoneal macrophages were pre-treated with goat anti-mouse Tim-4 antibody (Cat# AF2826, R&D,USA) or control goat IgG (R&D,USA) at 5 μg/ml for 1 h before stimulation with LPS (final concentration was 100 ng/ml) or 100 U/ml IFN-γ for the time indicated. For another two groups, 83.3 μg/ml of NF-κB inhibitory ligand (Cat# 25–007, upstate, USA) [15] together with 100 ng/ml LPS was added into cells for indicated time after pretreatment with anti-Tim-4 antibody or IgG.

RAW264.7 cells (ATCC TIB-71) were grown in Dulbecco’s Modified Eagle’s medium (DMEM, GIBCO, Grand Island, NY, USA) supplemented with 10% fetal bovine serum (FBS, Gibco-BRL, USA), penicillin (100 U/ml) and streptomycin (100 μg/ml). We cloned the mouse Tim-4 from RAW264.7 macrophages under the GenBank number NM178759. Stable macrophage cell line overexpressing Tim-4 (RAW-pcDNA3-Tim-4) and corresponding control cell line (RAW-pcDNA3) were established by regular selection in 500 μg/ml G418 for 3–4 weeks after transfection. Supernatants or total cell lysates of RAW264.7-pcDNA3-Tim-4 and RAW264.7-pcDNA3 cells were collected separately after stimulation with 100 ng/ml LPS or 100 U/ml IFN-γ for indicated time. For another two groups, 83.3 μg/ml NF-κB inhibitory ligand was added together with 100 ng/ml LPS for indicated time.

### RT-PCR

Peritoneal macrophages were isolated from mice according to the methods described previously [14]. Cells were lysed and total cellular RNA was extracted using Trizol reagent (Invitrogen, Carlsbad, CA, USA). cDNA was synthesized from 5 μg total RNA with M-MLV Reverse Transcriptase (Invitrogen). The cDNA was used as a template for PCR using 2×Taq PCR MasterMix (Tiangen, Beijing, China) according to the manufacturer’s instructions. The following primers were used. Forward: 5′-CTACAGACATAGCCGTACTCA-3′, Reverse: 5′-GTCTTCATCATCCCTCCC-3′ (*Tim-4*); Forward: 5′-TGCGTGACATCAAAGAGAAG-3′, Reverse: 5′-TCCATACCCAAGAAG-3′ (*β-actin*). Parameters for PCR were as followings: 3 min pre-denaturation at 95°C; 30 cycles of 95°C denaturation for 45 sec, 57°C annealing for 45 sec, and 72°C extension for 1 min, followed by a final 10 min extension at 72°C.

### Assay of nitric oxide

The medium was used to detect the secretion of NO with Griess reaction kit (Beyotime, Bejing, China) according to previously described [16].

### Western blotting

Total cell lysates were prepared and protein concentration was determined by the bicinchoninic acid (BCA) protein assay (Beyotime, Beijing, China). Cell extracts were subjected to SDS-PAGE, transferred onto a polyvinylidene fluoride (PVDF) membrane (Millipore Co., Bedford, MA, USA). Membranes were incubated with primary antibody at a concentration of 2 μg/ml, overnight at 4°C. Abs against phospho-NF-κB p65 (Cat# 3031), NF-κB p65 (Cat# 8242), phospho-Stat1 (Tyr701) (Cat# 9171), phospho-Jak2 (Cat# 3771), Stat1 (Cat# 9172) and Jak2 (Cat# 3230) were obtained from Cell Signaling Technology (CST, USA). Ab against iNOS (Cat# ab15323) was obtained from Abcam (Cambridge, MA, USA). HRP-conjugated goat anti-rabbit IgG (Beijing Zhongshan Golden Bridge Biotechnology Co., Ltd., China) was added at a concentration of 0.8 μg/ml to the membranes for 1 h at room temperature. The membranes were then developed by ECL using SuperSignal West Pico Trial Kit (Pierce Biotechnology, Rockford, USA).

### Statistical analysis

Data are presented as the means ± SDs. Differences between 2 groups were evaluated with the Student’s *t* test. *p* < 0.05 was accepted as a significant criterion.

## Results

### IFN-γ stimulation enhances *Tim-4* mRNA expression in macrophages

**Fig 1:**
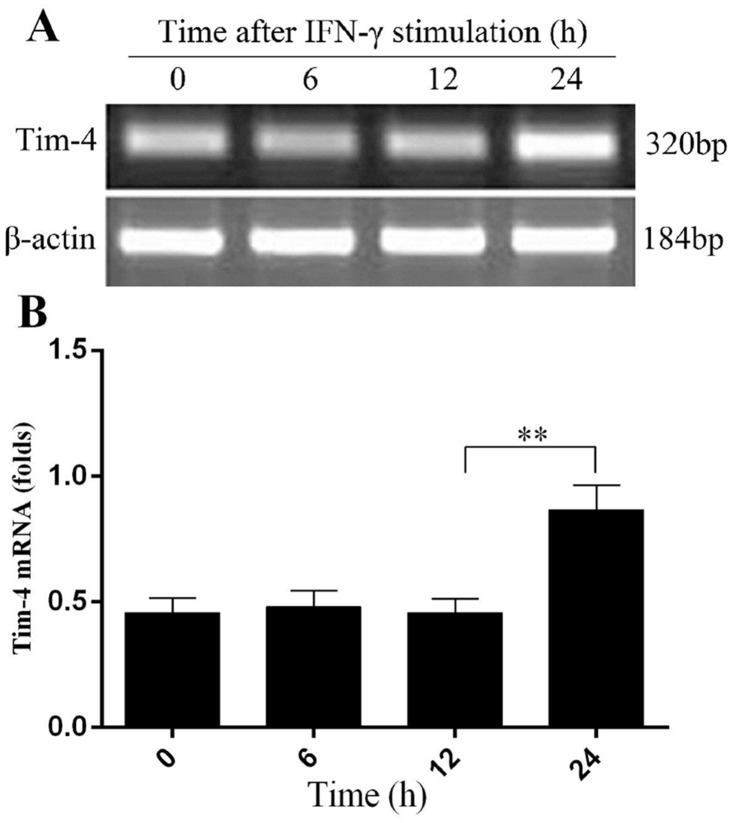
Up-regulation of *Tim-4* mRNA in peritoneal macrophages after IFN-γ stimulation. Peritoneal macrophages were isolated from BALB/c mice and then stimulated with 100 U/ml IFN-γ for the time indicated and *Tim-4* mRNA levels were determined by RT-PCR (A and B). In all panels, error bars represent the standard deviation from 4 independent cultures. A *p* value (student’s *t-*test) relative to the control cells of less than 0.01 is indicated by **.

Previously, we have shown that LPS promotes Tim-4 expression in a dose- and time- dependent manner [14]. To investigate the role of Tim-4 in NO production in activated macrophages, we further examined *Tim-4* mRNA expression in peritoneal macrophages in response to IFN-γ stimulation *in vitro*. A significant increase of *Tim-4* mRNA levels was found in peritoneal macrophages after incubation with 100 U/ml IFN-γ for 24 h (Fig 1A and 1B). These results indicated that Tim-4 expression could be up-regulated upon macrophage activation by IFN-γ.

### Tim-4 over-expression suppresses NO production and iNOS expression in LPS- or IFN-γ-activated macrophages

**Fig 2:**
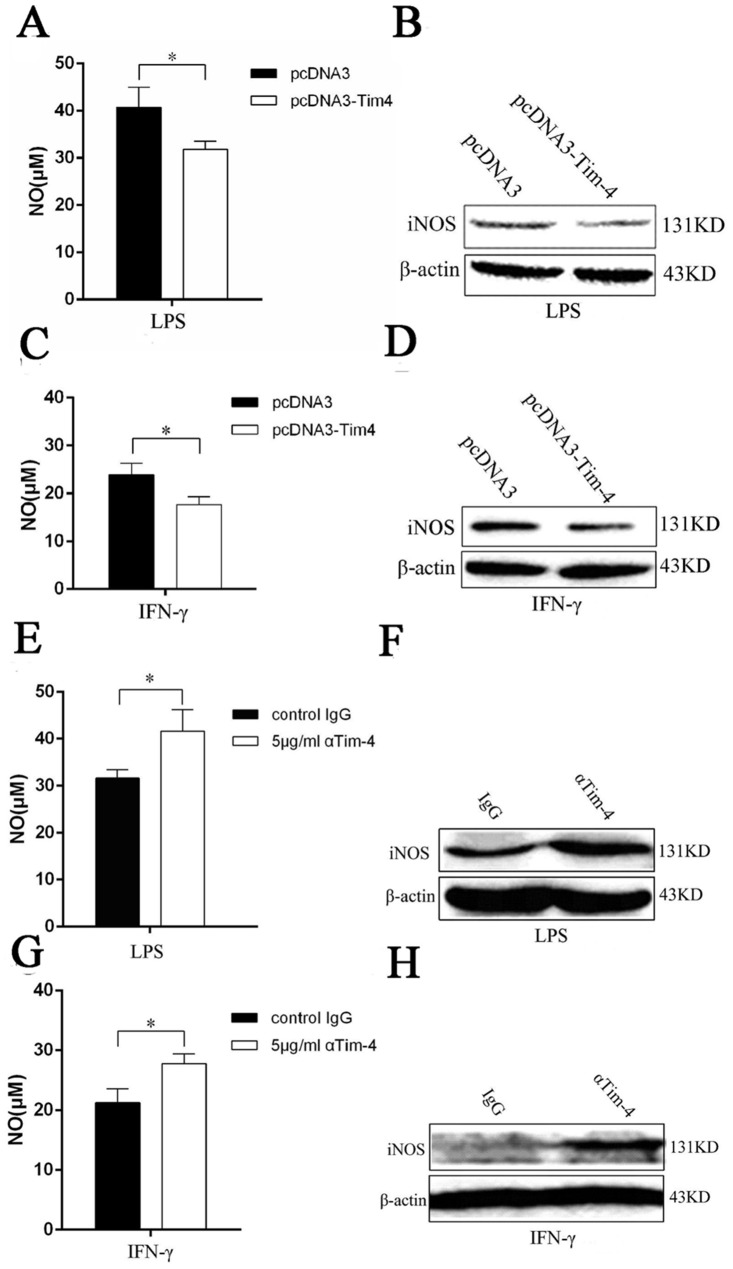
Tim-4 inhibited LPS/IFN-γ induced NO secretion and expression of iNOS protein. Tim-4 stably transfected cells RAW-pcDNA3-Tim-4 and control cells RAW-pcDNA3 were incubated with 100 ng/ml of LPS or 100 U/ml IFN-γ for indicated time. NO secretion in the supernatants was detected at 24 h after LPS or IFN-γ stimulation (A and C). iNOS expression was detected by western blot at 12 h after LPS (B) or IFN-γ treatment (D). Peritoneal macrophages were pre-treated with goat anti-mouse Tim-4 or control IgG at 5 μg/ml for 1 h, and then stimulated with 100 ng/ml LPS or 100 U/ml IFN-γ for 24 h. Supernatants were harvested for NO analysis (E and G). iNOS expression was detected by western blot at 12 h after LPS (F) or IFN-γ treatment (H). In all panels, error bars represent the standard deviation from 4 independent cultures. A *p* value (student’s *t-*test) relative to the control cells of less than 0.05 is indicated by *.

NO is known to be mainly secreted by activated macrophages. To verify whether Tim-4 takes effect on NO production of macrophages, stable cell lines RAW-pcDNA3-Tim-4 and RAW-pcDNA3 were stimulated with 100 ng/ml LPS or 100 U/ml IFN-γ and NO production was examined by Griess reaction. We found that compared to RAW-pcDNA3 cells, NO production in RAW-pcDNA3-Tim-4 cells was significantly downregulated after stimulation with LPS (Fig 2A) and IFN-γ (Fig 2C) for 24 h. Moreover, the levels of iNOS protein expression in RAW-pcDNA3-Tim-4 cells were also significantly lower than those of RAW-pcDNA3 cells after LPS or IFN-γ stimulation (Fig 2B and 2D) for 12 h. These results indicated that Tim-4 played an inhibitory role in NO production of macrophages.

### Tim-4 blockade promotes NO production and iNOS expression in LPS- or IFN-γ-activated macrophages

To further elucidate the negative regulation of Tim-4 on the production of NO in macrophages, peritoneal macrophages were pretreated with Tim-4 blocking antibody or isotype-matched control IgG, followed by the LPS or IFN-γ stimulation. We found that the levels of NO from peritoneal macrophages treated with anti-Tim-4 blocking antibody were significantly higher than those of isotype-matched control group (Fig 2E and 2G). To further investigate the effects of Tim-4 on NO production, we assayed the levels of iNOS protein expression by peritoneal macrophages with anti-Tim-4 treatment. Compared to control group, Tim-4 blockage induced higher iNOS expression in peritoneal macrophages with LPS or IFN-γ stimulation (Fig 2F and 2H). Collectively, Tim-4 blockade promoted LPS/IFN-γ-induced NO production of peritoneal macrophages, which further indicated that Tim-4 indeed inhibited NO production in activated macrophages.

### Tim-4 inhibits NO production in LPS-activated macrophages by NF-κB pathway

**Fig 3:**
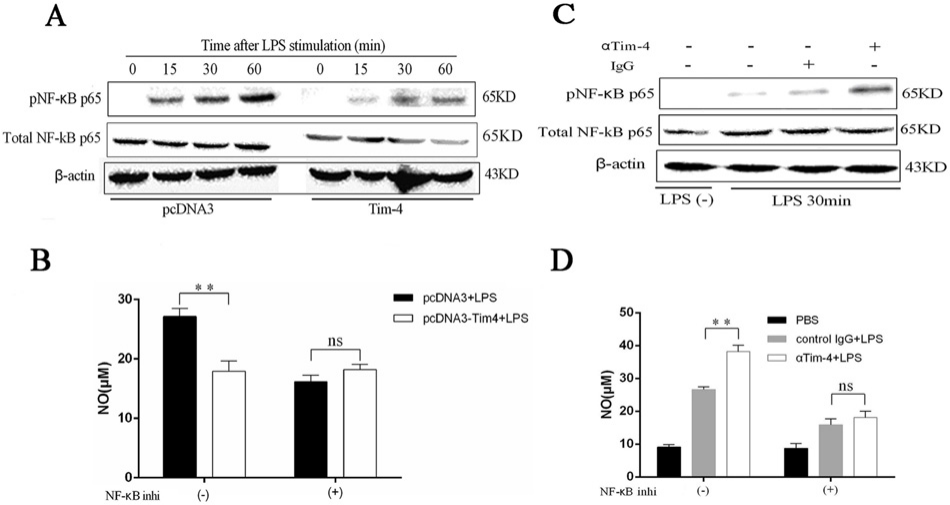
Tim-4 inhibited NO production by NF-κB signaling pathway. RAW-pcDNA3-Tim-4 and RAW-pcDNA3 cells were stimulated with LPS for the times indicated and western blot was used to detect phosphorylation of NF-κB p65 (A). Production of NO in LPS-activated RAW264.7 was detected before or after treatment with NF-κB inhibitory ligand (B). Peritoneal macrophages were pre-treated with goat anti-mouse Tim-4 antibody or control IgG at 5 μg/ml for 1 h, and then stimulated with 100 ng/ml LPS for 30 min. Cells were harvested for western blot (C). Production of NO in LPS-stimulated peritoneal macrophages with or without anti-Tim-4 pretreatment was detected before or after application of NF-κB inhibitory ligand (D). These experiments were repeated at least 3 times. Error bars represent the standard deviation from 4 independent cultures from 4 mice. A *p* value (student’s *t-*test) relative to the control cells of less than 0.01 is indicated by **. ns represents a *p* value greater than 0.05.

To clarify the signaling pathways involved in Tim-4-mediated negative regulation on NO production in macrophages, RAW-pcDNA3-Tim-4 and RAW-pcDNA3 cells were stimulated with LPS and western blot was used to evaluate the activation of NF-κB pathway, which was the classical pathway involved in NO secretion. The result showed that Tim-4 over-expression decreased phosphorylation of NF-κB p65 compared to the control group (Fig 3A). In order to address the critical role of NF-κB in Tim-4 mediated regulation on NO production, NF-κB inhibitory ligand was included to incubate with RAW-pcDNA3-Tim-4 and RAW-pcDNA3 cells before LPS stimulation. Results showed that NF-κB inhibitory ligand significantly decreased NO secretion (Fig 3B). Furthermore, down-regulation of NO induced by Tim-4 over-expression was abrogated by NF-κB inhibitory ligand in LPS stimulated macrophages (Fig 3B).

To further investigate the signaling pathways of Tim-4-mediated regulation on NO production in macrophages, peritoneal macrophages pretreated with Tim-4 blocking antibody were stimulated with LPS and western blot was used to assay NF-κB pathway. Consistent with the result of Tim-4 over-expression, Tim-4 blockade enhanced phosphorylation of NF-κB p65 at 30 min (Fig 3C). Moreover, up-regulation of NO induced by Tim-4 blockade was also abrogated by NF-κB inhibitory ligand in LPS stimulated macrophages (Fig 3D), suggesting that Tim-4 regulate NO production in LPS activated macrophages by NF-κB signaling pathway.

### Tim-4 inhibits Jak2-Stat1 pathway in IFN-γ-activated macrophages

**Fig 4:**
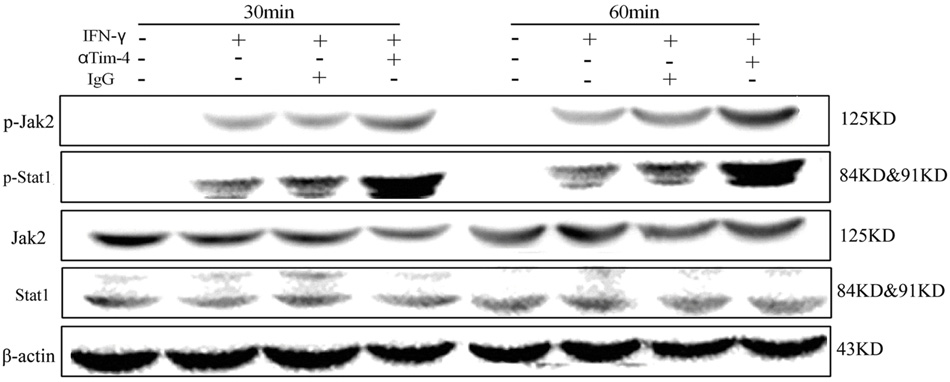
Tim-4 blocking promoted activation of Jak2-Stat1 pathway. Peritoneal macrophages were isolated from BALB/c mice and incubated with goat anti-mouse Tim-4 antibody or control IgG at 5 μg/ml for 1 h, then stimulated with 100 U/ml IFN-γ for 30 min or 60 min. The levels of phosphorylated Stat1 and Jak2 were determined by immunoblotting. These experiments were repeated at least 3 times.

To investigate the mechanism of Tim-4 mediated regulation on NO production in IFN-γ-activated macrophages, murine peritoneal macrophages pretreated with 5 μg/ml anti-Tim-4 blocking antibody were stimulated with IFN-γ. Enhanced phosphorylation of Jak2 and Stat1 was found in Tim-4 blocking group at 30 min and 60 min time points compared to the control group (Fig 4), indicating that Jak2-Stat1 pathway might be involved in Tim-4 mediated regulation on NO production in IFN-γ-activated macrophages. However, further studies need be performed to identify this issue.

## Discussion

Data reported here demonstrate that Tim-4 suppresses the production of NO in macrophages induced by LPS *via* inhibition NF-κB pathway. Our results also demonstrate that Tim-4 inhibits NO production, iNOS expression and Jak2-Stat1 signaling pathway induced by IFN-γ. This finding provides a hitherto unrecognized function of Tim-4, a molecule with potentially regulatory roles. This observation suggests that up-regulation of Tim-4 might be involved in the pathogenesis of macrophage mediated inflammation and autoimmune disease by inhibiting NO production.

NO is a pleiotropic signaling molecule and is involved in a variety of biological functions, including vascular relaxation, microvascular blood flow and permeability, platelet aggregation, neurotransmission, and immunomodulation [17–20]. It is also associated with the pathogenesis and control of infectious diseases, tumors, autoimmune processes such as rheumatoid arthritis, diabetes, and systemic lupus erythematosus (SLE) [21]. Macrophage derived NO, which is catalysed by iNOS, is a major source of immunomodulation in rodents [22]. Macrophages are well known to exert their functions in the status of activation through expressing co-stimulators and cytokines. Tim-4 is exclusively expressed on macrophages and mature DCs, and has been regarded as a new co-stimulator in immunity. So far, many studies of Tim-4 mainly focus on macrophage mediated phagocytosis of apoptotic cells and the regulation of T cell functions [9, 10, 12, 13, 23–25]. Our previous results indicate that Tim-4 is also involved in regulation of co-stimulator expression and cytokine production in macrophages [14]. So we speculate that Tim-4 might play key roles in NO modulation. To investigate the regulation of Tim-4 on macrophage derived NO, we used LPS and IFN-γ, which are the strong activators of iNOS, to stimulate macrophages. Our data show that Tim-4 could inhibit LPS- or IFN-γ-induced NO secretion and iNOS expression in murine macrophages by over-expression or blocking assay (Fig 2). These results further support the point that Tim-4 plays an important role in negative modulation of macrophage activity [14]. Renee M. Hein et al found that administration of rmTIM-1/his could increase NO production in macrophage cell line RAW264.7 [11]. Based on our data reported here, we speculate that rmTIM-1/his might promote RAW264.7 cells to produce NO by blocking Tim-4 mediated suppression of NO production. Of course, whether rmTIM-1/his induces NO production by the way other than Tim-4 requires further investigation.

In this paper, our findings coincide with the results that the expression of Tim-4 is increased in macrophages after LPS stimulation [14, 26, 27]. However, our results showed that over-expression of Tim-4 down-regulated iNOS protein expression and NO secretion in LPS or IFN-γ-activated macrophages (Fig 2A–2D), indicating that Tim-4 up-regulation might act as a mechanism of negative feedback regulation in activated macrophages. This result is consistent with that Tim-4 is essential for the maintenance of the homeostatic state of resident peritoneal macrophages [9]. Previously, we have demonstrated that Tim-4 overexpression decreases the expression levels of MHCII, CD80 and CD86 molecules, as well as TNF-α production [14], which is consistent with that Ly6C-CD169+ macrophages express higher levels of Tim-4 but lower of CD86 and MHCII [28]. These data indicate that RAW-pcDNA3-Tim-4 cells, which express higher levels of Tim-4, perform immune regulatory functions. Consistently, Tim-4 overexpression inhibiting NO production and iNOS expression further verified this point in the present study.

It is reported that M2-like tissue-resident macrophages express high levels of Tim-4 [28]. In the present study, we used starch induced peritoneal macrophages for Tim-4 antibody blockade assay, while the macrophage cell line RAW264.7 was used for overexpression. We detected the phenotypes of these cells by analyzing the ratio of Ly6C-CD11b+F4/80+ cells, which are regarded as the tissue resident macrophages [28]. As shown in S1 Fig, 72.8% peritoneal macrophages were Ly6C-CD11b+F4/80+ cells and 49.5% RAW264.7 cells were Ly6C-CD11b+F4/80+ cells, suggesting that most cells belong to tissue resident macrophages. However, whether Tim-4 is involved in maintaining the M2 phenotype requires to be clarified in the future.

However, the functions of NO are complex and diverse in health and disease. NO may be pro- or anti-inflammatory depending upon the disease state [29]. Previously, we reported that Tim-4 attenuates concanavalin A-induced hepatitis by regulating macrophages [14] and increased expression of Tim-4 is found in peripheral blood mononuclear cells from patients with SLE [30]. We speculate that Tim-4 might suppress the bactericidal response of macrophages by inhibiting NO production since Tim-4 contributes to the efficient cell-to-cell spread by L. monocytogenes in macrophages *in vitro* and the growth of these bacteria is impaired in Tim-4(-/-) mice [31]. However, the role of Tim-4 mediated regulation on NO of macrophages *in vivo* needs to be further investigated.

It is well known that NF-κB is an important transcription factor that plays a critical role in LPS-induced macrophage activation and production of proinflammatory cytokines and NO [32, 33]. In this study, the phosphorylation of NF-κB p65 induced by LPS was inhibited in macrophages with Tim-4 over-expression, while its phosphorylation was promoted in cells with Tim-4 blockade. What’s more, the NF-κB inhibitory ligand reversed the regulatory effect of Tim-4 on NO production (Fig 3). Based on these data, we confirmed that Tim-4 could inhibit NF-κB signaling pathway which was involved in Tim-4 mediated regulation on NO production in LPS-activated macrophages. For IFN-γ-induced NO production, the present study demonstrated that Tim-4 blocking could enhance IFN-γ-induced Jak2 and Stat1 phosphorylation (Fig 4). Whether Tim-4 reduces IFN-γ-induced NO production *via* Jak2/Stat1 pathway in macrophages requires to be verified. However, the mechanisms of Tim-4 suppressing NF-κB or Jak2/Stat1 pathway are not clear.

It is well known that Tim-4 does not contain a tyrosine-kinase phosphorylation motif in the cytoplasmic domain, indicating that Tim-4 could not complete signal transduction by itself [10]. Very recently, it is reported that Tim-4 utilizes integrins as co-receptors to effect phagocytosis of apoptotic cells [34]. In this process, Tim-4-driven phagocytosis depends on the activation of integrins and involves Src-family kinases, FAK, phosphatidylinositol 3,4,5-trisphosphate, nucleotide-exchange factor Vav3, as well as Rho-family GTPases. Furthermore, RGD-peptide lunasin inhibits Akt-mediated NF-κB pathway in human macrophages by interaction with the αVβ3 integrin [35]. It is reported that butein inhibits the LPS-induced increase of NO production and activation of iNOS gene expression by increasing the expression of αvβ3 integrin [36]. Since IgV region of Tim-4 contains RGD domain recognized by integrins, we speculate that αvβ3 integrin might be a signaling way for Tim-4 to inhibit NO production of macrophages. Tim-4 is well known as PS receptor and mediation the phagocytosis of apoptotic cells by macrophages [12, 13]. PS is largely confined to the inner leaflet of the plasma membrane of healthy cells, and PS exposure on the cell surface indicates apoptosis. However, transient PS translocation to the cell surface not only has been observed in sperm capacitation, myotube formation, phagocytosis, neutrophil stimulation [37], PS exposure but also has been related to evasion mechanisms of parasites known as apoptotic mimicry. NO production by activated macrophages is inhibited after infection with the PS^+^ subpopulation of Toxoplasma gondii [38]. It is not clear whether LPS or IFN-γ stimulation would induce PS exposure on macrophages. In addition, Tim-4 activates autophagy-mediated degradation of ingested tumors by directly interacting with AMPKα1 [39]. Recently Strom TB group reports that TIM-4hiCD169+ macrophages in mice are highly susceptible to apoptosis, while CD169+ tissue-resident macrophages are resistant to oxidative stress-induced apoptosis in mice lacking TIM-4 [28]. So we could not rule out the possibility that Tim-4 might decrease NO production partially by affecting macrophage viability after LPS or IFN-γ stimulation. However, whether these mechanisms involve in Tim-4 mediated inhibition of NO production by NF-κB or Jak2/Stat1 pathway requires further investigation in the future.

In conclusion, the present study showed that Tim-4 significantly inhibits NO production in murine macrophages activated by LPS/IFN-γ. Although more macrophage cell lines should be included in this study and further studies are needed to explain the detailed mechanism of Tim-4 mediated regulation on NO production, our study suggests that Tim-4 may contribute to its immune modulation by regulating NO secretion and might act as a novel potential target for treating inflammatory or autoimmune diseases.

## Supporting Information

S1 Fig The phenotype of peritoneal macrophages and RAW264.7 cells. (TIF)
