# Supplementary figures and images for: Tim-4 Inhibits NO Generation by Murine Macrophages
Source: PLoS One. 2015 Apr 23;10(4):e0124771. doi: 10.1371/journal.pone.0124771 (PMC4408120; doi:10.1371/journal.pone.0124771)

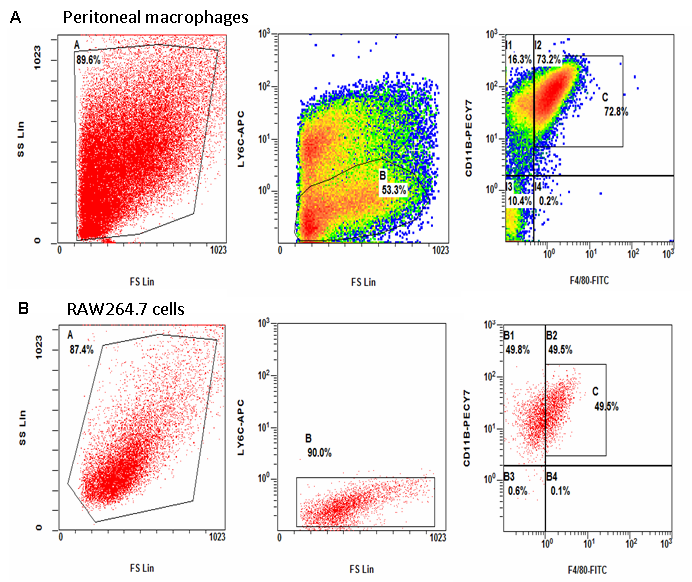

Supplement: S1 Fig — (TIF) [file pone.0124771.s001.tif]
